# Japan's health system toward 2040: structural challenges and a renewed social contract

**DOI:** 10.1016/j.lanwpc.2026.101920

**Published:** 2026-07-15

**Authors:** Lisa Yamasaki, Haruka Sakamoto, Hideki Hashimoto, Naoki Kondo, Osamu Kunii, Yusuke Tsugawa, Sarah Krull Abe, Satoshi Ezoe, Shinichi Egawa, Renzo R. Guinto, Ataru Igarashi, Angkana Lekagul, Kaung Suu Lwin, Mitsuru Mukaigawara, Ho Namkoong, Yoshitaka Nishino, Santosh Kumar Rauniyar, Eiko Saito, Kenji Shibuya, Keizo Takemi, Kun Tang, Takahiro Tabuchi, Hana Tomoi, Tomoko Suzuki, Shuhei Nomura

**Affiliations:** a Department of Environmental Health, Harvard T.H. Chan School of Public Health, Boston, MA, USA; b Department of Emergency Medicine and Critical Care, Japan Institute for Health Security, Tokyo, Japan; c Graduate School of Public Health, St Luke's International University, Tokyo, Japan; d Department of Health and Social Behavior, School of Public Health, Graduate School of Medicine, The University of Tokyo, Tokyo, Japan; e Department of Social Epidemiology, Graduate School of Medicine and School of Public Health, Kyoto University, Kyoto, Japan; f Global Health Innovative Technology Fund (GHIT Fund), Tokyo, Japan; g Division of General Internal Medicine and Health Services Research, David Geffen School of Medicine at UCLA, Los Angeles, CA, USA; h Economic Research Institute for ASEAN and East Asia (ERIA), Jakarta, Indonesia; i Department of Global Health Policy, Graduate School of Medicine, The University of Tokyo, Tokyo, Japan; j Nagasaki University, Nagasaki, Japan; k International Cooperation for Disaster Medicine Lab., International Research Institute of Disaster Science (IRIDeS), Tohoku University, Sendai, Japan; l SingHealth Duke-NUS Global Health Institute, Duke-NUS Medical School, National University of Singapore, Singapore; m Graduate School of Pharmaceutical Sciences, The University of Tokyo, Tokyo, Japan; n International Health Policy Program Foundation (IHPP Foundation), Nonthaburi, Thailand; o University of Huddersfield, Huddersfield, England, UK; p Department of Government, Harvard University, Cambridge, MA, USA; q Department of Infectious Diseases, Keio University School of Medicine, Tokyo, Japan; r Japan Center for International Exchange (JCIE), Tokyo, Japan; s Ocean Policy Research Institute, The Sasakawa Peace Foundation, Tokyo, Japan; t Sustainable Society Design Center, Graduate School of Frontier Sciences, The University of Tokyo, Chiba, Japan; u Medical Excellence Japan, Tokyo, Japan; v Nagasaki University, The School of Tropical Medicine and Global Health, Nagasaki, Japan; w Vanke School of Public Health, Tsinghua University, Beijing, China; x Division of Epidemiology, School of Public Health, Tohoku University Graduate School of Medicine, Sendai, Japan; y The London School of Hygiene and Tropical Medicine, Department of Infectious Disease Epidemiology, Faculty of Epidemiology and Population Health, London, UK; z Global Health Policy Lab, International Research Institute of Disaster Science (IRIDeS), Tohoku University, Sendai, Japan; aa Department of Health Policy and Management, School of Medicine, Keio University, Tokyo, Japan; ab Department of Food, Nutrition and Health, Graduate School of International University of Health and Welfare, Tokyo, Japan

**Keywords:** Universal, health coverage, Health system reform, Japan, Demographic ageing, Value-based care, Health equity, Governance, Western Pacific

## Abstract

Japan's universal health coverage system has supported healthy longevity, but now confronts institutional stagnation: widely recognised structural pressures persist without reform of its core architecture. A multidisciplinary working group of 25 experts from Japan and the Western Pacific identified three interdependent structural challenges perpetuating policy inertia. These comprise a volume-driven, hospital-centric delivery model sustained by fee-for-service incentives, rigid budget allocation, and underdeveloped health technology assessment; fragmented data infrastructure and eroded public trust undermining evidence-informed governance; and institutional insularity limiting global interdependence. We propose a Vision 2040 framework with three goals: transitioning to an open, community-based ecosystem prioritising daily functioning and wellbeing; establishing trust and scientific independence as foundations of governance; and ensuring equity through circular innovation, in which Japan contributes to and learns from regional health networks by integrating global learning with domestic value assessment. Nine recommendations provide a roadmap for governance reform, data integration, reimbursement realignment with societal value, and reciprocal workforce partnerships. Reconstructing the social contract underpinning universal health coverage is central to sustainability across ageing Western Pacific societies.

## Introduction

Japan's healthcare system has long been recognised as a widely cited model for Universal Health Coverage (UHC), instrumental in achieving a society with healthy longevity.^1^ It now confronts structural pressures that challenge its foundational principles—universal access, equity, and financial risk protection. By 2040, more than one in three Japanese residents will be aged 65 and older,^2^ with healthcare and long-term care expenditures projected to reach historically unprecedented levels. Rapid population ageing and increasing fiscal constraints^3^^,^^4^ intersect with broader resilience risks shared across the Western Pacific, including natural disasters, emerging infectious diseases, and geopolitical instability. Together, these pressures challenge a delivery model designed for the disease patterns and social conditions of the 20th century, exposing growing tension within the implicit social contract underpinning Japan's post-war health system: the expectation that UHC can be sustained without fundamental redistribution of roles, risks, and responsibilities among government, providers, and households. As the burden of disease shifts decisively toward chronic conditions,^5^ multimorbidity,^6^ and integrated community-based care needs,^7^ the capacity of this legacy system to respond effectively is increasingly in question.

What Japan faces is not system failure, but institutional stagnation—where widely recognised structural pressures persist without corresponding recalibration of the system's core architecture. This stagnation reflects deep-seated political and economic constraints rather than technical barriers. These constraints originate in an entrenched institutional settlement: established stakeholder arrangements, a risk-averse political culture, and a successful track record of achieving healthy longevity with relatively low health expenditure render large-scale structural reforms, including fee schedule reconfiguration and acute-care bed consolidation, politically prohibitive.^8^^,^^9^ This is the dynamic of institutional path dependence, in which early institutional choices increasingly constrain the range of feasible reform options. These dynamics shift the financial burden onto vulnerable groups: low-income households and those with older members remain particularly exposed to catastrophic health expenditures, as current safety nets, including caps on out-of-pocket medical expenses, have proven inadequate.^10^ At its core, this is a question of who bears which risks—the substance of the social contract underpinning UHC. The same inertia that obstructs adaptation to domestic demographic shifts also constrains Japan's capacity to exercise credible leadership in global health governance, including in shaping norms on healthy ageing and UHC.

At the same time, intensifying cross-border interdependencies are reshaping the relationship between domestic and global health,^11^ yet Japan's institutions have not kept pace with these transformations. Cross-border patient flows^12^ and accelerating international migration of health professionals^13^ deepen interdependence among health systems worldwide. In Japan, however, these external shifts have not prompted the structural reforms required to align incentives and delivery capacity with population health needs, including payment system redesign, hospital bed consolidation, workforce reallocation, care integration, and health promotion. The result is a widening disconnect between global trends and domestic policy, in which intensifying interdependence is treated as an external diplomatic agenda rather than a resource for domestic reform.

Building on this disconnect, the analysis applies a circular innovation framework in which knowledge, technology, and human capital flow in both directions between domestic and global health systems.^14^ This framing departs from reverse innovation and south–south learning, which typically describe one-way flows between high- and low-income settings. The circular view fits Japan's current position as a mature high-income system facing demographic and fiscal pressures: Japan exports institutional knowledge such as long-term care insurance implemented in 2000, and has scope to import frontier practice such as community health worker models and digital care delivery developed by Western Pacific partners. This framework, developed in parallel with the companion paper on Japan's global health strategy,^15^ treats domestic reform and regional engagement as interdependent agendas rather than separated tracks, and grounds recommendations in governance and institutional analysis rather than technical convergence.

While recent initiatives, notably Japan's Third Healthcare Policy (the government's current five-year strategic plan for healthcare),^16^ acknowledge these challenges, the fundamental structural transformation required has yet to take hold. This study, informed by a multidisciplinary working group spanning academic research, policy practice, and civil society, articulates a new paradigm for Japan's national health strategy that combines resilient domestic system design with global interdependence as a foundation for sustainability. The analysis begins with a diagnosis of structural challenges perpetuating policy inertia, builds toward a Vision 2040 grounded in a renegotiated social contract, and concludes with a roadmap of actionable interventions that move Japan from current constraints to future resilience, with circular innovation informing engagement across regional health systems. Japan's trajectory therefore serves as a test case for societies confronting analogous pressures, with lessons of regional and global relevance.

## Methods

### Study design and participant group

A multidisciplinary working group of 25 members was convened to identify structural challenges in Japan's health system and develop recommendations for reform. Members were drawn from Japan and other countries across the Western Pacific and beyond, representing academic research, policy practice, and civil society. The group included early-career professionals alongside senior figures.

### Expert survey and thematic analysis

The expert consultation was conducted in two sequential rounds and informed the development of the study's conceptual framework (Supplementary Method). In Round 1, we identified structural challenges in Japan's health system. In Round 2, informed by the thematic synthesis of Round 1, we articulated forward-looking goals and recommendations linked to those challenges. Responses were analysed using inductive thematic analysis,^17^ through six iterative stages: familiarisation, initial coding generated directly from the data, theme generation, theme review, theme definition and naming, and assessment of thematic convergence. We then discussed the consolidated themes and their interpretation to reach consensus. The themes spanned governance, financing, data infrastructure, workforce, global engagement, and equity, and were integrated into the three structural challenge categories, three strategic goals, and nine recommendations presented in this study. Policy options that did not emerge as priority themes fall outside this framework but remain legitimate avenues for future policy deliberation. Throughout, the structural challenges were identified from survey-derived themes and are supported by an extensive body of existing research. The diagnosis of structural challenges leads into a Vision 2040 built around three strategic goals, followed by a roadmap of nine recommendations grounded in the diagnosis and the circular innovation framework.

### Search strategy and selection criteria

The literature search was designed as an integrative review to support and contextualise the findings of the working group. We searched PubMed and Ichushi-Web (Japan Medical Abstracts Society) for articles published between January 1, 2000, and January 15, 2026. Search terms covered the core themes of this paper, including “health system reform”, “universal health coverage”, “healthy longevity”, “population ageing”, “long-term care”, “health workforce”, “foreign healthcare workers”, “health financing”, “health governance”, “primary care”, “health equity”, “sustainability”, and “social contract”. We focused on peer-reviewed articles and policy analyses. The database search was supplemented by hand-searching reference lists of key reviews. We also conducted targeted searches of official policy documents and grey literature from government agencies, intergovernmental bodies, and health diplomacy forums in the Western Pacific. Publications in English and Japanese were reviewed to capture both international and Japanese-language sources. Two authors independently screened titles and abstracts, with disagreements resolved through discussion. We included sources judged relevant and informative for the aims of this study.

### Role of the funding source

The funders had no role in the design of the review, the analysis or interpretation of data, the writing of the manuscript, or the decision to submit for publication.

## The anatomy of policy paralysis

We identified three interdependent structural challenges that create a reinforcing cycle of policy inertia associated with structural misalignment (Table 1). First, a systemic mismatch persists in which a predominantly fee-for-service architecture, sustained by established stakeholder interests, may incentivise high-volume services over patient-centred outcomes. Second, limitations in evidence generation and data linkage constrain the system's capacity to evaluate effectiveness and address social determinants. Third, institutional arrangements may limit engagement with global talent and mutual understanding on healthcare governance. These are not only technical gaps, but also institutional and governance barriers rooted in historical path dependence—whereby early institutional choices constrain subsequent reform options^18^^,^^19^—that collectively constrain reforms needed for sustainability. Each challenge amplifies the others: misaligned incentives generate inefficiencies that fragmented data cannot expose, while institutional insularity prevents external learning that might disrupt entrenched arrangements. The following subsections examine each challenge in detail.

**Table 1:** Key structural challenges of Japan's health system.

Structural challenge category	Specific critical challenges
A. Systemic mismatch with a new era of health challenges	**Incentive misalignment:** Reform momentum is constrained by established stakeholder arrangements that impede reimbursement restructuring, perpetuating a system originally designed for 20th-century disease patterns. **Structural overuse:** Fee-for-service incentives may encourage high-volume “low-value care” (e.g., unnecessary antibiotic prescriptions for upper respiratory infection) rather than patient value, with estimated inefficiency of approximately 1.6–1.9% of total healthcare spending. **Neglected equity:** Absence of standardised monitoring for financial protection masks regressive cost burden on low-income households and those with older members, potentially undermining the principle of equal treatment for equal need. **Allocation rigidity:** Professional boundaries block necessary task-shifting, creating a functional physician shortage by burdening doctors with delegable or automatable tasks; bed consolidation under the Regional Medical Care Vision remains politically stalled.
B. An outdated infrastructure inhibiting a paradigm shift	**Evidence deficit:** Policy evaluation is often based on ad-hoc use of administrative statistics that can rationalise political inertia rather than stimulate critical and deliberative policy discussion; the absence of explicit logic models linking policy inputs to health and social outcomes allows ineffective programmes to persist. **Data fragmentation:** Persistent gaps in linking clinical data with welfare and long-term care records limit visibility into Social Determinants of Health (SDOH), perpetuating biomedical individualism. **Unfiltered technology adoption:** Technology adoption proceeds through automatic public insurance coverage upon regulatory approval without separate value-based evaluation or mechanisms to de-implement low-value legacy practices; HTA remains institutionally underdeveloped, largely confined to marginal price adjustment rather than binary coverage decisions or systematic de-implementation of low-value care. **Trust deficit:** Weak risk communication infrastructure leaves the system vulnerable to misinformation and public distrust, as demonstrated by the prolonged HPV vaccine policy vacuum (2013–2022).
C. Insularity and underutilisation of global interdependence	**Partial integration:** Foreign worker numbers have increased, yet workers are incorporated through a fragmented patchwork of visa schemes prioritising short-term labour supplementation over professional settlement, equitable inclusion, and system learning, limiting qualitative integration. **Corporatisation risk:** Limited international engagement overlooks global warning signs regarding healthcare corporatisation and its implications for equity, cost escalation, and workforce burnout. **Reform constraints:** Change-oriented advocacy coalitions remain fragmented and lack sustained organisational capacity, while short electoral cycles limit incentives to pursue reforms whose benefits materialise only over the medium term. **Asymmetric workforce flows:** Recruitment strategies risk unsustainable talent outflows from neighbouring countries without ensuring reciprocal skill transfer, circular migration frameworks, or regional health security coordination.

*Abbreviations:* SDOH = Social Determinants of Health; HTA = Health Technology Assessment.

Three interdependent structural challenges were identified through the survey analysis; each reinforces the others, collectively perpetuating policy inertia.

### Systemic mismatch with a new era of health challenges

**Fig. 1:**
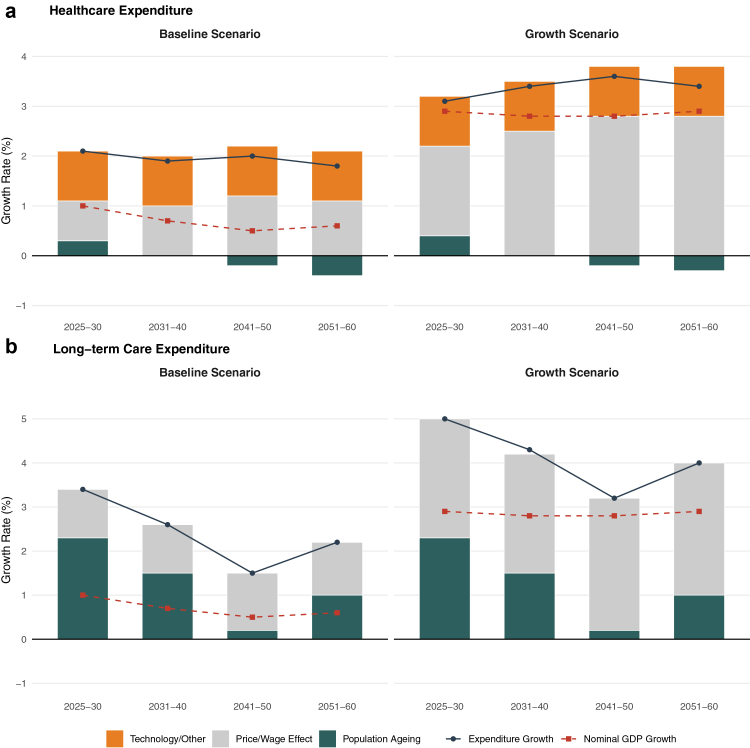
**Decomposition of healthcare and long-term care expenditure growth in Japan by cost driver (adapted from Cabinet Office projections).** This figure reformats, for clarity of presentation, the decomposition of projected healthcare and long-term care expenditure growth in Japan originally published by the Cabinet Office in its long-term fiscal projections.^20^ The decomposition separates expenditure growth into three components: demographic effect (population ageing), price and wage effect, and technology and other factors. Panel (a) displays healthcare expenditure and panel (b) displays long-term care expenditure growth rates (%, annual average). Two macroeconomic scenarios are shown: Baseline Scenario (Total Factor Productivity [TFP] growth 0.5%, left panels) and Growth Scenario (TFP growth 1.1%, right panels). Stacked bars represent each component's contribution to expenditure growth; solid lines indicate total expenditure growth and dashed lines indicate nominal GDP growth. The gap between expenditure growth and GDP growth reflects the fiscal pressure on social security financing. The data show that technology-related factors drive healthcare cost growth, whereas demographic change primarily drives long-term care costs, underscoring that undifferentiated fiscal debates obscure distinct policy challenges requiring tailored responses.

**Fig. 2:**
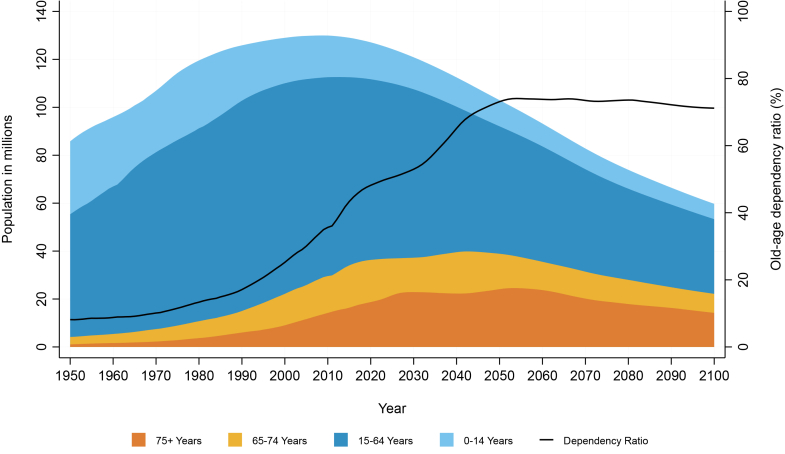
**The demographic squeeze: diverging population cohorts and old-age dependency ratios in Japan (1950–2100).** Historical and projected population of Japan, stratified by age category: 0–14 years (light blue), 15–64 years (blue), 65–74 years (yellow-orange), and 75 years and older (red-orange). Data from 1950 to 2017 represent historical estimates; data from 2018 to 2100 represent projections. The superimposed black line represents the old-age dependency ratio (right axis), calculated as population aged 65 and older divided by working-age population (15–64 years). This visualisation illustrates structural inversion: rapid working-age population contraction shrinks the tax base precisely as the cohort aged 75 and older, which has the highest healthcare and long-term care utilisation, enters substantial growth, creating a dependency ratio that incremental fee-schedule adjustments alone cannot resolve.

Japan's health system exhibits a persistent structural mismatch between contemporary disease burden and legacy fiscal-reimbursement architecture. Policy debates often focus on aggregate fiscal balance, with limited differentiation between fundamentally distinct drivers of health expenditure. The Cabinet Office of Japan has decomposed projected expenditure growth into demographic, price, and technology-driven components (Fig. 1), showing that technology-related factors constitute the dominant determinant of medical spending growth while population ageing primarily drives long-term care costs—a distinction that current policy frameworks inadequately address.^20^ Fig. 2 illustrates the substantial shift in demographic assumptions underpinning Japan's social security system. The trajectory reveals not merely population ageing but a fundamental structural inversion: rapid contraction of the working-age population (15–64 years) shrinks the tax base precisely as the high-utilisation cohort aged 75 and older enters substantial growth. This demographic squeeze creates a dependency ratio that incremental fee-schedule adjustments alone cannot resolve.^21^

*Data source:* Cabinet Office, Government of Japan (Economic and Social Research Institute; Econometric Analysis Division). Long-term projections of the economy, public finance, and social security (Cabinet Office estimates) [Japanese]. 2024. https://www.esri.cao.go.jp/jp/esri/workshop/forum/241004/pdf/241004_siryo01.pdf (accessed May 16, 2026).

*Data source:* Global Fertility, Mortality, Migration, and Population Forecasts 2017–2100. Institute for Health Metrics and Evaluation (IHME). 2020. https://ghdx.healthdata.org/record/ihme-data/global-population-forecasts-2017-2100 (accessed May 1, 2026).

The current reimbursement architecture creates substantial misalignment between provider solvency and the quality and efficiency of care delivery. While originally designed to ensure universal access, the fee-for-service incentive structure now tends to produce a “high-volume, low-margin” business model. Although partial countermeasures exist—including prospective payment through the Diagnosis Procedure Combination (DPC) system, bundled payments for chronic disease management, and monthly reimbursement caps—none has fundamentally altered volume-driven incentives.^22^ Because the government strictly controls unit prices while leaving quantity largely flexible—apart from bundled payments—providers may face pressure to maximise service volume for financial viability. This dynamic contributes to the proliferation of “low-value care,” including unnecessary antibiotic prescriptions for upper respiratory infections, where reimbursement structures shape clinical decision-making in ways that do not necessarily improve patient outcomes.^23^ This model also creates financial disincentives for productivity: by linking revenue to service volume rather than outcomes, it discourages efforts to streamline care through innovation or task-shifting. Claims-based analyses applying internationally established evidence to identify low-value services estimate that eliminating such inefficiencies, together with greater use of generics and biosimilars, could reduce national medical expenditure by 1.6–1.9%.^24^

This system-side inefficiency shifts part of the burden of sustainability to households. Despite protections such as caps on out-of-pocket medical expenses, current mechanisms fail to shield vulnerable households from combined medical and long-term care costs, disproportionately burdening low-income households with older members,^25^ who face high and potentially catastrophic out-of-pocket payments that undermine the principle of equal treatment for equal need.

**Fig. 3:**
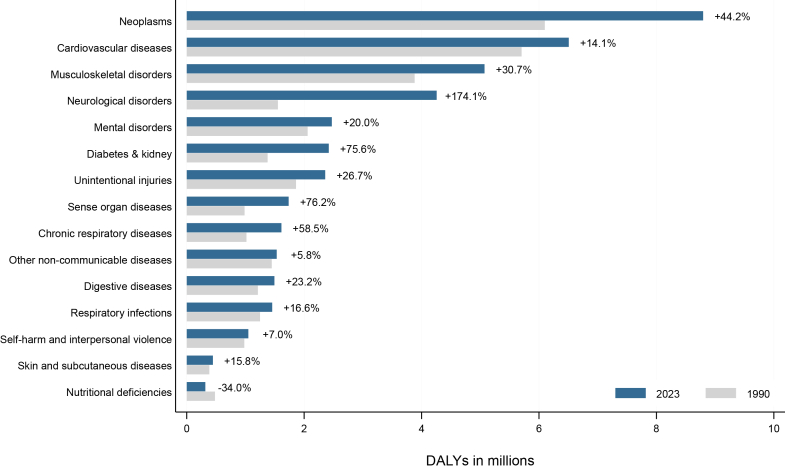
**The epidemiological transition: change in all-age DALYs by major cause in Japan (1990 vs. 2023).** Absolute disease burden measured in Disability-Adjusted Life Years (DALYs) for the top 15 Level 2 cause categories (broad disease groupings such as cardiovascular diseases, neoplasms, and neurological disorders as classified in the Global Burden of Disease Study hierarchy), compared between 1990 (grey bars) and 2023 (blue bars). Causes are ranked by total DALYs in 2023. Percentage values indicate relative change in DALYs from 1990 to 2023. While neoplasms and cardiovascular diseases remain significant in volume, neurological disorders (+174.1%) and diabetes and kidney diseases (+75.6%) exhibit the most rapid expansion, reflecting a structural shift in health needs from conditions requiring acute intervention toward those demanding long-term care management.

The prevailing institutional logic reinforces this misalignment. Incentives are tightly coupled to an acute, hospital-centric, organ-specific model of care delivery, which may perpetuate services whose clinical value has not been systematically assessed within existing accountability frameworks.^22^ This bias is reflected in the hospital landscape: Japan has one of the highest total hospital bed densities among Organisation for Economic Co-operation and Development (OECD) nations, with approximately 12 beds per 1000 population (including both acute and medical long-term care beds within hospitals),^26^ yet many beds designated as “acute” effectively serve sub-acute or long-term care functions, as the reimbursement structure incentivises hospitals to maintain high occupancy rates to sustain revenue.^27^ Meanwhile, incentives for longitudinal multimorbidity management and community-based care remain critically insufficient given the epidemiological transition. Fig. 3 illustrates the mismatch between Japan's established medical infrastructure and its accelerating epidemiological reality. Analysis of disease burden trends from 1990 to 2023 reveals a notable divergence: while the burden from conditions historically dominating acute care—measured in disability-adjusted life years (DALYs)—increased in absolute terms due to population ageing, their relative growth was moderate for neoplasms (+44.2%) and cardiovascular diseases (+14.1%).^28^ By contrast, drivers of disability and complex long-term care needs increased markedly, with neurological disorders (including dementia) increasing 174.1% and diabetes and kidney diseases rising 75.6%.^28^ This trajectory illustrates a deepening systemic misalignment: a health system historically optimised for “cure” of acute events faces growing “care” demands from chronic, degenerative conditions defined by frailty and cognitive decline.

*Data source:* GBD Results. Institute for Health Metrics and Evaluation (IHME). 2025. https://vizhub.healthdata.org/gbd-results/(accessed May 1, 2026).

Reform constraints persist because this configuration remains entrenched within reimbursement negotiations and hospital workforce structures. The limited progress of the “Regional Medical Care Vision”—a government initiative launched in 2014 requiring each prefecture to develop plans for optimising hospital bed distribution—illustrates the political difficulty of consolidating bed functions: despite a decade of planning, actual bed reductions have fallen far short of targets,^29^ reflecting the complexity of coordinating multiple stakeholders—including hospitals, local governments, and medical associations—each with distinct operational priorities and institutional constraints. This allocative rigidity extends to human resources: strict professional boundaries limit necessary task-shifting, contributing to a functional physician shortage by assigning physicians tasks that could be delegated to other professionals or automated.^30^

### An outdated infrastructure inhibiting a paradigm shift

Structural transformation of Japan's health system is constrained by persistent deficiencies in core institutional infrastructure, most notably in data integration, evidence generation, trust governance, and economic value assessment. Despite advances in digital technology, health-related data in Japan remain highly fragmented across medical, welfare, and long-term care sectors.^31^ These domains are administered through institutionally siloed systems with separate databases that cannot be readily linked at the individual level, limiting the system's capacity to generate actionable evidence and evaluate policy effectiveness beyond narrow clinical outcomes. These constraints primarily reflect governance failures—rather than technological limitations.^31^ The persistence of these deficiencies, despite longstanding recognition, may reflect underlying incentive structures: standardisation and integration impose immediate administrative costs on operational agencies, whereas benefits accrue diffusely over the medium term.^32^ This fragmentation obscures regional and institutional disparities in healthcare resource allocation, quality, and outcomes. It also perpetuates a form of “biomedical individualism” that renders social determinants of health systematically invisible, preventing analysis of how factors such as income, housing, and social isolation shape health outcomes.^33^ Consequently, the system bears the high medical costs of unaddressed social determinants without possessing the data infrastructure to identify root causes,^34^ potentially contributing to patterns of overinvestment in clinical remediation and underinvestment in housing, income security, and social connectivity.

The COVID-19 pandemic exposed the extent of this infrastructural weakness.^35^ The initial reliance on fax-based reporting and the uneven deployment of HER-SYS (Health Center Real-time Information-sharing System on COVID-19)—hampered by duplicate data entry, poor interoperability with electronic medical records, and inconsistent adoption across municipalities^35^—revealed the absence of unified health data architecture capable of supporting timely decision-making. More fundamentally, policy evaluation in Japan's health sector often relies on ad-hoc administrative statistics that describe existing conditions rather than critically assessing policy effects—for example, fee-schedule revisions are assessed through descriptive summaries without scrutiny of whether reforms achieve their intended outcomes, limiting accountability and institutional learning. The absence of explicit logic models linking policy inputs to health and social outcomes allows ineffective programmes to persist unchallenged.

In parallel, institutional capacity for risk communication and trust governance remains underdeveloped. Recent health crises and rapid misinformation diffusion have underscored public trust as a core function of modern health systems. The prolonged suspension of Japan's active recommendation for human papillomavirus (HPV) vaccination (2013–2022)—during which the government ceased proactive outreach while maintaining programme availability—illustrates this trust deficit: policy responses followed public anxiety rather than epidemiological evidence, resulting in prolonged policy inaction and avoidable cancer risks.^36^ Japan's initial COVID-19 vaccine rollout was among the slowest in the OECD, owing to cautious regulatory approval; early-phase public hesitancy was documented in parallel,^37^ reflecting trust-sensitive governance dynamics.^38^ Mechanisms for independent scientific communication and proactive trust-building remain underdeveloped and poorly embedded in routine governance structures.

These failures converge in a persistent innovation-sustainability dilemma. While Japan rapidly adopts new medical technologies through its system of automatic public insurance coverage upon regulatory approval—meaning new drugs and devices become reimbursable without separate value-based evaluation—mechanisms to evaluate value comprehensively or to de-implement low-value legacy practices remain politically fragile. Health Technology Assessment (HTA), which should provide an objective framework for assessing clinical benefit, cost-effectiveness, affordability, and broader societal value, remains institutionally underdeveloped in contrast to more institutionalised frameworks in several OECD countries.^39^ In particular, its use has been largely confined to marginal price adjustment rather than to binary coverage decisions or systematic de-implementation of low-value care.^40^ Without transparent and independent value assessment embedded within robust institutional safeguards, and insulated from industry influence and short-term policy incentives favouring visible innovation, the system cannot reliably distinguish productive investment from low-value or inefficient spending. This dynamic may perpetuate uncritical technology adoption and, over time, erode both fiscal sustainability and public trust. The roots of this gap lie in HTA's institutional design. In Panel 1, we provide a literature-informed structural analysis of Japan's HTA system, focussing on comparator specification bias, over-reliance on single-metric evaluation, and the policy implications of these design choices.

Panel 1 **Structural challenges in Japan's HTA: implications for value, uncertainty, and innovation.**

• Japan's health technology assessment (HTA) system is formally organised around cost–effectiveness analysis, yet its operational design differs fundamentally from value-based appraisal models such as the National Institute for Health and Care Excellence (NICE). A defining feature is the near-mechanical translation of incremental cost-effectiveness ratios (ICERs) into price revision rates.^41^ Because ICER estimates are highly sensitive to comparator choice, subgroup specification, and modelling assumptions, this linkage transmits analytical uncertainty directly into reimbursement outcomes, producing large and sometimes disproportionate price adjustments from fragile evidence.
• Comparator specification is a major source of structural bias. Japanese appraisals often restrict analysis to a single low-cost comparator irrespective of real-world treatment pathways,^41^ whereas NICE typically adopts mixed or practice-reflective comparators and considers treatment displacement.^42^ This methodological difference predisposes Japanese evaluations toward findings of “no additional benefit,” effectively converting HTA into a cost-minimisation exercise rather than a multidimensional value assessment. The result is an apparent technocratic neutrality masking ex-ante value compression.
• A second limitation is the implicit reliance on a single metric to represent complex value. Attempts to collapse clinical benefit, caregiver burden, productivity, equity, and unmet need into a single cost-per-quality-adjusted life year (QALY) threshold assume the existence of a “Super QALY,” which is conceptually untenable.^43^ Quantitative indicators are necessary but cannot substitute for deliberative appraisal processes that integrate guideline design, analytical framing, critical review, and contextual judgement. Treating ICER thresholds as decision rules rather than inputs obscures uncertainty and narrows the evaluative space.
• The appraisal of disease-modifying therapies for dementia illustrates the importance of analytical perspective. Although long-term care costs are conceptually acknowledged in Japan, modelling rules can minimise caregiver quality-of-life effects, preventing family spillovers from influencing ICERs.^39^ NICE, by contrast, separates perspectives—healthcare payer for costs and a broader outcome perspective—thereby permitting caregiver QALYs in the base case and recognising the relational burden of dementia.
• These structural features have three policy implications. First, ICER-to-price algorithms amplify rather than manage uncertainty. Second, comparator restriction and QALY centrality risk systematic under-recognition of innovation. Third, process design—transparency, practice-reflective comparators, explicit uncertainty handling, and multi-component value frameworks—is as important as the metric itself. Without such reforms, HTA in Japan will remain a post-hoc price adjustment tool rather than a mechanism capable of balancing fiscal sustainability, equitable access, and credible innovation incentives.

### Insularity and underutilisation of global interdependence

Japan's health policy remains largely insular, with limited use of global interdependence as a resource for domestic reform. Global health continues to be positioned primarily as an external or diplomatic domain rather than as part of domestic health system reform and resilience. This framing may constrain the system's capacity to learn from and integrate with regional and global health networks despite intensifying demographic pressures and workforce shortages.

Despite recent reforms signalling recognition of foreign workers as longer-term contributors, Japan's historically fragmented and scheme-based approach has risked prioritising short-term labour supplementation over systemic integration. Without reciprocal frameworks for professional recognition and mutual capacity-building, workforce inflows risk falling short of the principles of the World Health Organization Global Code of Practice on the International Recruitment of Health Personnel.^44^ This insularity does not result from inaction. Foreign workers employed in Japan's health and care sectors increased steadily over the past decade (Supplementary Figure S1), reflecting growing reliance on international labour to sustain service delivery. The 2024 legislation formalising this transition (effective April 2027)^45^ further codified this policy trajectory.

However, this quantitative increase has not yet fully translated into qualitative integration. Foreign health workers have historically been incorporated through multiple policy schemes—including bilateral Economic Partnership Agreements, the specified skilled worker programme, and the former technical internship system—that have prioritised short-term labour supplementation over long-term system resilience (Supplementary Table S1).^46^^,^^47^ Workforce inflows expanded without a commensurate strategy for professional settlement, equitable inclusion,^48^ and system learning. Japan's non-ratification of the UN International Convention on the Protection of the Rights of All Migrant Workers underscores the structural barriers to expanding reciprocal frameworks. Implementation gaps risk constraining opportunities for organisational learning, skill diffusion, and feedback of new practices into the domestic system. Without reciprocal frameworks, recruitment strategies risk reproducing extractive dynamics that weaken regional health security rather than reinforcing it.^49^

Beyond questions of institutional design, the political dynamics of reform remain structurally constrained. Change-oriented advocacy coalitions—comprising patient advocates, public health and policy researchers, innovative providers, and fiscal policymakers—remain fragmented and lack sustained organisational capacity, while short electoral cycles limit incentives to pursue reforms whose benefits materialise only over the medium term. As a result, political incentives to reconfigure established arrangements remain modest. Japan's health system faces challenges in internalising lessons from comparable health systems facing similar demographic and fiscal pressures, including experiences with healthcare corporatisation and its implications for equity, cost escalation, and workforce burnout. Together, these dynamics limit Japan's ability to use the global interdependence required to navigate demographic decline and sustain an equitable health system.

## Vision 2040: reconstructing the social contract

Drawing on priorities articulated through our survey analysis, the proposed reform agenda represents a fundamental departure from the legacy of the 1961 universal health insurance system. That system—a closed, hospital-centric model—was historically significant but has proven increasingly difficult to sustain in a super-aged society characterised by longevity and frailty. The transformation we propose rests on three strategic pillars that define Japan's future health architecture: (1) progressive transition from a volume-driven, hospital-centric model—characterised by over-utilisation of both inpatient and outpatient services—to an open, community-based ecosystem that prioritises daily functioning and wellbeing alongside clinical care; (2) building trust and scientific independence as essential infrastructure for health governance; and (3) realisation of health equity through circular innovation, where global learning and rigorous domestic value assessment work in concert to ensure fair access to effective interventions. Table 2 summarises these three goals and their corresponding action domains. Together, these pillars seek to reconstruct the social contract, shifting the framing of the health system from a fiscal constraint to a resilient foundation for social and economic stability. The following sections examine each goal.

**Table 2:** Goal–action matrix for health system transformation.

Goals/Actions	1. Governance reform: enhancing coordination and strengthening accountability	2. Data and technology implementation: ensuring trust, quality, and interoperability	3. Resource reallocation: investing in sustainability and communities
Goal 1: Transition from hospital-centric to an open, community-based model	• Redefine public-private mix: Guarantee essential benefit package for equity while granting outcome-based accountability for lifestyle support services • Establish operational guidelines empowering welfare and housing sectors to prevent medicalisation of social issues • Institutionalise social prescribing with strict non-medical governance to bridge clinical care with community support	• Deploy human-centric digital tools to complement home-based care and automate administrative tasks • Build unified data infrastructure anchored on functioning for daily living beyond the medical model • Deepen integrated care via real-time information sharing across health and social sectors	• Establish reciprocal training partnerships positioning Japan as an Asian hub for care workforce development, prioritising skills transfer over temporary labour recruitment • Invest in intergenerational communities to mitigate social isolation and reduce long-term care dependency • Induce cross-sector collaboration by making it a prerequisite for government subsidies
Goal 2: Build trust and scientific independence as essential infrastructure for health governance	• Embed behavioural science-based risk communication into government crisis management to counter polarisation • Define misinformation as a national security risk and launch multi-sectoral monitoring systems • Institutionalise scientific advice via an independent scientific advisory body legally separated from political decision-making	• Deploy human-centric Unified ID to visualise daily living functions and automate administrative burdens, exposing hidden disparities • Mandate data governance compliance and transparency as requirement for reimbursement • Build intelligence systems to monitor climate and health risks in integrated manner	• Introduce civic education curricula to cultivate scientific reasoning and fact-checking skills (scientific citizenship) • Build public trust in government and experts through routine, deliberative citizen dialogue • Permanently incorporate deliberative citizen processes into policy evaluation to ensure democratic legitimacy
Goal 3: Ensure equity through circular innovation and global integration	• Revise reimbursement based on societal value metrics (e.g., DALYs, caregiver burden, productivity loss) to prioritise high-value interventions • Mandate full disclosure of government-industry interactions to strengthen accountability mechanisms and ensure patient-centred policy • Establish statutory mechanisms to identify and delist low-value interventions from the public reimbursement package	• Publicly evaluate healthcare providers using standardised metrics for clinical outcomes and cost-efficiency • Visualise budget priorities using disease burden metrics like DALYs to correct allocative inefficiency • Harmonise regulations and create procurement pathways to actively import reverse innovation from Asia	• Restore redistribution functions by shielding households from catastrophic expenditure and rejecting regressive burden shifts • Secure revenue by strengthening taxation on health-harming products (e.g., tobacco) and earmarking it for prevention and health promotion • Shift government focus from process micromanagement to outcome verification, enabling private innovation

*Abbreviations:* DALYs = Disability-Adjusted Life Years; ID = Identification.

Rows represent the three strategic goals and columns represent three domains of policy action derived from the survey. Each cell lists recommendations that advance the corresponding goal within that action domain.

### Goal 1: transition from hospital-centric to an open, community-based model

The health system of a super-aged society must move beyond organisation around episodic acute care. Japan should shift toward an open, community-based ecosystem that prioritises daily functioning and wellbeing alongside clinical intervention—aligning with the principles articulated in the Ottawa Charter for Health Promotion and the life-course approach to health.^50^ This transformation seeks to address the “medicalisation of social issues”—a structural tendency where health consequences of social determinants, such as prescribing sleeping pills for insomnia rooted in social isolation, receive pharmaceutical treatment without subsequent action to address the underlying social determinants.^51^ In this future state, social prescribing functions as a core element of community health, with welfare and housing sectors—not medical hierarchies—playing leading governance roles under transparent outcome monitoring and equity safeguards. Implementation must be accompanied by rigorous evaluation frameworks to establish the evidence base for non-clinical interventions. While a diverse market of lifestyle support services flourishes beyond the essential benefit package, safeguards should protect against commercial determinants of health that can undermine population wellbeing.^52^

### Goal 2: build trust and scientific independence as essential infrastructure for health governance

The COVID-19 pandemic revealed substantial trust deficits in Japan's health governance. Trust is a core determinant of health-system resilience, and once depleted, it narrows the range of interventions a system can implement effectively.^53^ A defining feature of renewed governance is structural separation of scientific expertise from political decision-making: while elected leaders retain authority to weigh broader societal considerations, system design should ensure political choices are not presented as scientific conclusions. Citizens participate in deliberative processes that cultivate “scientific citizenship”^54^—the capacity to engage critically with evidence and distinguish reliable information from misinformation. Data infrastructure functions as an equity backbone, making disparities affecting marginalised populations visible and addressable, while integrated intelligence monitors emerging threats including climate-health risks.

### Goal 3: ensure equity through circular innovation and global integration

Achieving allocative efficiency depends on credible value assessment. As the structural analysis of Japan's HTA system demonstrates (Panel 1), current mechanisms for value assessment remain inadequate for this transformation. Financial sustainability requires a shift from cost containment toward allocative efficiency based on societal value. This shift acknowledges that in a labour-short society, keeping patients in the workforce and relieving family caregivers represent core value components alongside clinical outcomes.^55^ Resource allocation should reflect actual disease burden rather than historical spending patterns. Simultaneously, Japan should embrace a “mutual learning stance” replacing previous unilateral approaches to global health. The system should actively absorb cost-effective innovations developed in resource-constrained settings—from community health worker programmes grounded in primary healthcare principles of community autonomy and local participation to mobile diagnostics—while contributing its own expertise in geriatric care to regional partners. This circular model could transform a demographic challenge into a shared regional opportunity. A forthcoming paper develops this circular co-evolution framework in the global context, proposing a strategic pivot toward human security diplomacy that links Japan's domestic reform with regional partnership across the Western Pacific.^15^

## From diagnosis to action: a strategic roadmap

Building on the three strategic goals outlined above, we propose a roadmap to overcome the political inertia of the 1961 regime (Table 2). The roadmap is organised by three action domains—governance reform, data and technology implementation, and resource reallocation—each of which operationalises all three strategic goals presented above. Table 2 sets out the full goal–action matrix, with each cell elaborated in the Recommendations that follow. These recommendations call for a shift from bureaucratic micromanagement and siloed operations to a dynamic model defined by value-based transparency, evidence-informed governance, and circular resource allocation. Critically, past reform proposals have often stalled not for lack of technical merit but because the political costs of structural change were concentrated on well-organised incumbent interests while benefits remained diffuse and long-term; our roadmap therefore addresses implementation incentives alongside policy design. Operationalising these interventions would help break the cycle of structural misalignment and shift the framing of the health system from a fiscal constraint to a resilient foundation for social stability. Realising this roadmap requires a new political coalition; reformers should mobilise the business sector and civil society to counterbalance established professional interests that have historically constrained structural change.

### Recommendation 1: reform governance to enable value-based healthcare

Governance reform provides the institutional foundation for all subsequent recommendations. Three interconnected changes are needed: redefining the public-private boundary, institutionalising scientific independence, and mandating transparency.

#### Redefine the public-private division and shift to outcome-based accountability

This recommendation involves shifting from detailed, process-based regulation of healthcare delivery to systems that grant providers greater autonomy while maintaining accountability for outcomes. Government continues to guarantee an essential benefit package: a standardised set of publicly financed core medical and long-term care services that ensures equity and universal access. Beyond this guaranteed core, non-public providers—including private clinics, for-profit care facilities, and commercial health service companies—should be granted greater flexibility to innovate in delivering lifestyle support services that help individuals maintain functional ability and daily wellbeing outside fee schedules, subject to rigorous outcome evaluation and regulatory safeguards ensuring that commercial incentives do not override patient welfare.^56^ Cross-ministerial policy guidance should clarify that welfare and housing sectors take primary responsibility for addressing social determinants of health, allowing medical institutions to focus on clinical care.

Regulatory oversight by prefectural and national authorities should move from prescriptive input requirements toward outcome verification, and should consider de-implementation of “low-value” commercial services^57^ that prioritise high-margin pharmaceutical interventions over cost-effective preventive care.^52^ This would enable providers to optimise service delivery based on patient outcomes, quality of life, and cost-efficiency while maintaining equity safeguards.

#### Institutionalise scientific independence and protect information integrity

An independent scientific advisory body, legally distinct from political decision-making and complemented by civic education programmes that cultivate scientific citizenship, should be established to ensure evidence-informed responses to health crises. Legislation mandating this independence is essential for preserving the integrity of scientific advice regardless of short-term political pressures.^58^ Protecting public trust also requires treating health misinformation as a critical infrastructure risk. Behavioural science-informed risk communication^59^ should be integrated into government crisis management. Coordinated monitoring across health, media, and security agencies—working together to detect and respond to health-related misinformation and disinformation—should treat information integrity as vital infrastructure essential for national security.^60^

#### Mandate transparency in policy-making and provider performance

Full public disclosure of all interactions between public officials and pharmaceutical, medical device, and healthcare industry representatives should be mandated. Such transparency would mitigate informal lobbying influence and help ensure that fee-schedule revisions—which determine reimbursement rates for medical services—prioritise patient benefit over the financial interests of hospitals, physician associations, or industry. Policy evaluation using reimbursement claims data should operate under clear rules governing data access and require open reporting of analytical results, enabling independent verification of reform impacts.^61^ At the institutional level, hospitals and clinics should be required to publicly report standardised clinical outcome metrics and cost-efficiency measures. This would help identify low-value care while encouraging practices that improve patient outcomes. Together, these mechanisms shift the locus of accountability from industry and provider associations back to patients and taxpayers.

### Recommendation 2: build integrated data infrastructure to enable value-based resource allocation

Japan's health-related data remain trapped in institutional silos that systematically obscure social determinants and lock resource allocation into volume-driven patterns. Constructing a unified data infrastructure—a digital backbone for value-based resource allocation—is a prerequisite for the transformation envisioned above.

#### Link health and social data to capture functional status and identify disparities

Linking medical, long-term care, and welfare data through the expanded national identification system would enable monitoring of daily living functions beyond clinical metrics. Achieving this linkage requires overcoming the political incentive barriers identified earlier. Strategies may include incorporating data interoperability requirements into reimbursement criteria, establishing independent data governance frameworks that separate technical oversight from political intervention,^61^ and creating visible pilot projects whose demonstrable outcomes help build political support and sustain momentum for broader reform.^62^ By revealing patterns of service utilisation, unmet need, and outcome variation across socioeconomic strata, such integrated data infrastructure would enable systematic identification of health disparities affecting marginalised populations—including migrant workers and isolated older adults—that traditional siloed systems overlook.^63^

Interoperability standards should facilitate real-time information sharing across health and social sectors, ensuring interventions address root causes of vulnerability rather than symptoms alone. Complementing this infrastructure, government investment should prioritise digital tools that automate administrative tasks, allowing healthcare professionals to focus on face-to-face care rather than administrative documentation. The academic community can support this infrastructure through methodological innovation, open data practices, and sustained engagement with policymakers.

#### Realign reimbursement with societal value and disease burden

Realigning reimbursement requires revising pricing schedules based on societal value assessment incorporating caregiver burden, productivity effects, and quality-of-life dimensions, not solely clinical efficacy. Visualising budget priorities using multidimensional metrics such as DALYs would help identify areas where spending patterns diverge from actual disease burden, enabling more equitable resource allocation. A statutory body should be empowered to identify and manage low-value interventions within public packages. At the institutional level, transparency initiatives should evaluate individual hospitals and clinics using standardised metrics for clinical outcomes and cost-efficiency,^64^ referencing global models including the UK's Patient-Reported Outcome Measures (PROMs) collection^65^ and US Medicare's Hospital Compare system,^66^ encouraging transition from volume-driven competition to value-driven accountability.

#### Develop predictive analytics for climate-health risks

Health threats posed by climate change—from heat- and typhoon-related health outcomes to infectious disease outbreaks—will increasingly interact and compound, requiring integrated surveillance that links environmental and health data.^67^ The data infrastructure described in the sections ‘Link health and social data to capture functional status and identify disparities’ and ‘Realign reimbursement with societal value and disease burden’ provides the foundation for such monitoring. Using linked health and social records, predictive analytics platforms can identify populations and individuals at heightened risk—such as isolated older adults living in energy-poor housing—potentially enabling proactive outreach before climate-related health events occur.^68^ This application of data infrastructure illustrates how investments in digital health could yield both routine efficiency gains and enhanced preparedness for emerging threats.

### Recommendation 3: reallocate resources toward community-based care and global integration

Strategic resource reallocation should proceed along two complementary tracks. Domestically, integrating social prescribing across health, long-term care, and welfare sectors would redirect resources toward prevention and community-based support. Internationally, Japan should actively learn from other Asian countries facing similar demographic challenges. This includes adopting cost-effective innovations from resource-constrained settings and establishing reciprocal training partnerships that position Japan as a hub for regional workforce development.

#### Integrate social prescribing and incentivise cross-sector collaboration

Integrating social prescribing into national care pathways would bridge clinical care with community support.^69^ The long-term care insurance system, currently focused on physical and cognitive function assessment, should be expanded to evaluate and support social participation. Existing community comprehensive support centres, already mandated to coordinate services for older adults, could be expanded and empowered to ensure referrals effectively address social causes of distress. Existing programmes that already address social dimensions, including community-based psychiatric rehabilitation and employment support in cancer and cardiovascular care, should be expanded as models for broader social prescribing implementation. Public subsidies should incentivise demonstrated cross-sector collaboration, encouraging medical, welfare, and housing providers to integrate operations. Capital investment should be directed toward intergenerational communities^70^ designed to mitigate social isolation, shifting resources from treating downstream consequences to building upstream community resilience.

#### Adopt innovations from Asia and strengthen fiscal foundations for prevention and equity

Japan's regulatory frameworks have evolved in relative isolation from international standards, limiting opportunities for cross-border learning. Harmonising domestic regulations with international benchmarks would facilitate adoption of reverse innovation from other Asian health systems. Procurement pathways should be developed to actively import innovations from resource-constrained settings—such as community health worker programmes for chronic disease management and mobile health platforms for remote monitoring—to address domestic workforce shortages and rural access gaps.

Adopting innovations from abroad should be matched by strengthening Japan's domestic fiscal foundations to safeguard equity and finance prevention. Redistribution functions should be reinforced to protect households from catastrophic health expenditure.^71^ Revenue from taxation on health-harming products—including tobacco—should be legally earmarked for reinvestment in prevention and health promotion, creating a sustainable funding stream for upstream interventions.^72^ Such earmarked investment must prioritise structural and environmental interventions over individually focused health education.

#### Build reciprocal workforce partnerships, strengthen deliberative processes, and invest in civic education

Japan must move beyond short-term labour augmentation and reposition itself as a regional talent mobility hub for the care workforce in the Western Pacific. This requires reorienting workforce mobility away from temporary labour substitution toward reciprocal and circular partnerships that prioritise qualification reciprocity, skills-sharing, and long-term career development. Such arrangements would allow cross-border mobility to contribute not only to domestic workforce sustainability but also to capacity-building in source countries, including Thailand, Viet Nam, the Philippines, and Indonesia, enabling human capital flows to reinforce, rather than undermine, regional health security. Despite recent reforms and expanded workforce inflows, current approaches remain primarily oriented toward meeting immediate domestic labour demand, with limited institutionalisation of circular arrangements that translate mobility into system learning and innovation within the care sector.^73^ A forthcoming paper further examines how regional digital interoperability frameworks and mutual recognition of professional qualifications can operationalise this circular workforce mobility across the Western Pacific.^15^ Embedding circular human capital principles—alongside sector-specific settlement pathways and equitable cost-sharing for worker mobility—would allow Japan to move beyond labour supplementation and function as a platform for high-standard care workforce development, including in geriatric care and digital health.

Durable implementation of these reforms, however, depends on more than institutional design. Workforce integration intersects with immigration attitudes, professional boundaries, and community cohesion—domains in which policy sustainability requires informed public consent.^74^ Policy evaluation should therefore incorporate deliberative citizen processes, including citizens' assemblies, to support societal learning from implementation and to build democratic legitimacy for structural change. Underpinning these deliberative mechanisms, publicly financed civic education should cultivate scientific literacy and fact-checking skills. Evidence-informed strategies such as early misinformation awareness^75^ can strengthen citizens' capacity to engage constructively in health policy debates, respond critically to misinformation and disinformation, and participate meaningfully in shaping the future of Japan's health and care systems.

## Conclusion

The future of Japan's health system lies not in preserving its legacy structure but in redesigning the social contract for a new era. Addressing institutional inertia would reframe rapid population ageing as a trigger for institutional renewal rather than a problem to contain, turning structural challenges into sources of resilience. Japan has an opportunity to develop circular innovation, in which domestic experience and frontier practice from regional partners reinforce each other. By taking this open and adaptive path, Japan could show that a mature society is defined less by the age profile of its population than by the resilience of its institutional design. Such reform would offer concrete experience for other rapidly ageing systems—neighbouring countries approaching the same demographic stage, source countries of health and care workers within the region, and larger economies facing similar pressures at greater scale. It would also highlight risks that any society navigating demographic transition may face: delayed delivery-system reform, fragmented data governance, and workforce strategies that do not embed reciprocal regional learning. As the frontrunner of this transition, Japan's choices today may shape policy trajectories for the region's health security in the coming decades.

## Contributors

SN conceptualised the review and developed the analytical framework. LY, HS, and SN developed the methodology and conducted the thematic analysis of the authors' responses to the expert consultation. LY, HS, and SN accessed and verified the underlying materials and data used in the analysis, including consultation responses, coded thematic outputs, and literature-derived evidence. LY and SN drafted the original manuscript. All authors contributed to the interpretation of findings and provided critical input on the analytical content. LY, HH, AI, and SN prepared the figures and tables. YN, TS, and SN coordinated project administration. SN supervised the review and acquired funding. All authors critically revised the manuscript for important intellectual content, approved the final version, and agree to be accountable for all aspects of the work. SN is the corresponding author.

## Declaration of generative AI and AI-assisted technologies in the writing process

During the preparation of this manuscript, the authors used Claude (Anthropic, San Francisco, CA, USA) in order to improve the readability and language of the manuscript. After using this tool, the authors reviewed and edited the content as needed and take full responsibility for the content of the publication.

## Declaration of interests

Keizo Takemi is chair of the Asian Population and Development Association and served as Japan's Minister of Health, Labour and Welfare from 2023 to 2024. Ataru Igarashi reports grants or contracts from Moderna and Takeda Pharmaceutical Company Limited, consulting fees from PhRMA Japan, EFPIA Japan, and the Japan Pharmaceutical Manufacturers Association, and honoraria from the Ministry of Health, Labour and Welfare of Japan, outside the submitted work. All other authors declare no competing interests. The views expressed in this Review are those of the authors and do not necessarily represent those of their institutions.

## Appendix A Supplementary data

Supplementary Material

Translated Abstract

## References

[1] Reich M.R., Ikegami N., Shibuya K., Takemi K. (2011). 50 years of pursuing a healthy society in Japan. Lancet.

[2] National Institute of Population and Social Security Research (IPSS) Population projections for Japan (2023 revision): 2021 to 2070. https://www.ipss.go.jp/pp-zenkoku/e/zenkoku_e2023/pp2023e_Summary.pdf.

[3] Ministry of Finance, Japan (2025). Japanese public finance fact sheet. https://www.mof.go.jp/english/policy/budget/budget/fy2025/02.pdf.

[4] OECD (2024). OECD economic surveys: Japan 2024. OECD. https://www.oecd.org/en/publications/oecd-economic-surveys-japan-2024_41e807f9-en.html.

[5] Nomura S., Murakami M., Rauniyar S.K. (2025). Three decades of population health changes in Japan, 1990–2021: a subnational analysis for the Global Burden of Disease Study 2021. Lancet Public Health.

[6] Saito Y., Igarashi A., Nakayama T., Fukuma S. (2023). Prevalence of multimorbidity and its associations with hospitalisation or death in Japan 2014–2019: a retrospective cohort study using nationwide medical claims data in the middle-aged generation. BMJ Open.

[7] OECD OECD reviews of public health: Japan | OECD. https://www.oecd.org/en/publications/oecd-reviews-of-public-health-japan_9789264311602-en.html.

[8] Katori T. (2024). Japan's healthcare delivery system: from its historical evolution to the challenges of a super-aged society. Glob Health Med.

[9] Ministry of Health, Labour and Welfare (2024). Challenges against Japanese UHC system. https://www.mhlw.go.jp/content/10500000/001463115.pdf.

[10] Hashimoto H., Tokunaga R. (2021). Household impact of medical and long-term care burden and burden fairness [Japanese]. Iryo To Shakai.

[11] Kickbusch I., Liu A. (2022). Global health diplomacy—reconstructing power and governance. Lancet.

[12] Lu J., Kubo S., Hashimoto M. (2024). The future of inbound medical care as gauged from the foreigners undergoing complete medical examinations in Japan. Glob Health Med.

[13] Social Welfare and War Victims’ Relief Bureau, Ministry of Health, labour and welfare (MHLW), Japan (2024). Current status and future directions for the acceptance of foreign care workers [Japanese]. https://www.mhlw.go.jp/content/12000000/001478533.pdf.

[14] Crisp N. (2014). Mutual learning and reverse innovation–where next?. Global Health.

[15] Sakamoto H, Yamasaki L, Abe SK (2026). Japan’s global health engagement in the Western Pacific: diagnosing structural inertia and a framework for human security diplomacy. Lancet Reg Health West Pac.

[16] Cabinet Secretariat, Government of Japan Healthcare and medical strategy (Third Term) [Japanese]. https://www.cas.go.jp/jp/seisakukaigi/kenkouiryou/tyousakai/dai43/gijisidai.html.

[17] Braun V., Clarke V. (2006). Using thematic analysis in psychology. Qual Res Psychol.

[18] Yamagishi T. (2022). Health insurance politics in Japan: policy development, government, and the Japan Medical Association. Cornell University Press. https://www.jstor.org/stable/10.7591/j.ctv1trhsqg.

[19] Mahoney J. (2000). Path dependence in historical sociology. Theory Soc.

[20] Cabinet Office, Government of Japan (Economic and Social Research Institute; Econometric Analysis Division) (2024). Long-term projections of the economy, public finance, and social security (Cabinet Office estimates) [Japanese]. https://www.esri.cao.go.jp/jp/esri/workshop/forum/241004/pdf/241004_siryo01.pdf.

[21] Ikegami N., Campbell J.C. (2004). Japan's health care system: containing costs and attempting reform. Health Aff.

[22] Hashimoto H., Ikegami N., Shibuya K. (2011). Cost containment and quality of care in Japan: is there a trade-off?. Lancet.

[23] OECD (2017).

[24] Tsugawa Y., Kato H., Igarashi A., Miyawaki A., Tamada Y., Goto R. (2025). Report on evidence required for optimizing healthcare expenditures [Japanese]. https://jhpra.jp/working_papers/.

[25] Shibuya K., Hashimoto H., Ikegami N. (2011). Future of Japan's system of good health at low cost with equity: beyond universal coverage. Lancet.

[26] OECD (2023).

[27] Jones R.S. (2009).

[28] Hay S.I., Ong K.L., Santomauro D.F. (2025). Burden of 375 diseases and injuries, risk-attributable burden of 88 risk factors, and healthy life expectancy in 204 countries and territories, including 660 subnational locations, 1990–2023: a systematic analysis for the Global Burden of Disease Study 2023. Lancet.

[29] Taneda K., Kakinuma T., Nakanishi Y., Kobayashi K., Akahane M. (2023). Community health care vision: toward realizing the desired medical service system. https://www.niph.go.jp/journal/data/72-1/202372010006.pdf.

[30] Koike S., Wada H., Ohde S., Ide H., Taneda K., Tanigawa T. (2024). Working hours of full-time hospital physicians in Japan: a cross-sectional nationwide survey. BMC Public Health.

[31] Hiramatsu K., Barrett A., Miyata Y. (2021). PhRMA Japan Medical Affairs Committee Working Group 1. Current status, challenges, and future perspectives of real-world data and real-world evidence in Japan. Drugs Real World Outcomes.

[32] Ikegami N., Campbell J.C. (2008). Dealing with the medical axis-of-power: the case of Japan. Health Econ Policy Law.

[33] Krieger N. (2001). Theories for social epidemiology in the 21st century: an ecosocial perspective. Int J Epidemiol.

[34] Bradley E.H., Elkins B.R., Herrin J., Elbel B. (2011). Health and social services expenditures: associations with health outcomes. BMJ Qual Saf.

[35] Takeshita K., Takao H., Imoto S., Murayama Y. (2022). Improvement of the Japanese healthcare data system for the effective management of patients with COVID-19: a national survey. Int J Med Inf.

[36] Hanley S.J.B., Yoshioka E., Ito Y., Kishi R. (2015). HPV vaccination crisis in Japan. Lancet.

[37] Okubo R., Yoshioka T., Ohfuji S., Matsuo T., Tabuchi T. (2021). COVID-19 vaccine hesitancy and its associated factors in Japan. Vaccines (Basel).

[38] Tomoi H., Hanley S.J.B., Larson H.J., Tomoi H., Masuda K. (2026). Determinants of human papillomavirus vaccination decision-making in Japan: a scoping review exploring contextual, social, and adolescent-specific influences. Vaccine.

[39] Shiroiwa T. (2020). Cost-effectiveness evaluation for pricing medicines and devices: a new value-based price adjustment system in Japan. Int J Technol Assess Health Care.

[40] Kamae I., Thwaites R., Hamada A., Fernandez J.L. (2020). Health technology assessment in Japan: a work in progress. J Med Econ.

[41] Hasegawa M., Komoto S., Shiroiwa T., Fukuda T. (2020). Formal implementation of cost-effectiveness evaluations in Japan: a unique health technology assessment system. Value Health.

[42] (2022). NICE technology appraisal and highly specialised technologies guidance: the manual. https://www.nice.org.uk/process/pmg36/chapter/evidence-2.

[43] Drummond M.F., Sculpher M.J., Claxton K., Stoddart G.L., Torrance G.W. (2015).

[44] World Health Organization (2024). WHO global code of practice: an overview. https://www.who.int/publications/m/item/who-global-code-of-practice--an-overview.

[45] Ministry of Justice, Government of Japan Overview of the development employment system (revised December 2025) [Japanese]. https://www.moj.go.jp/isa/content/001452485.pdf.

[46] Shoki R., Kono A., Hirano Y.O., Barroga E., Ota E., Nagamatsu Y. (2024). Factors related to job continuance of nurses who migrated to Japan: a cross-sectional study. Nurs Rep.

[47] Nagaya Y., Gillin N., Smith D. (2024). South-East Asian nurses' experiences under the Economic Partnership Agreement (EPA) in Japan: how language ability affects self-confidence and interpersonal relationships. J Transcult Nurs.

[48] Yasukawa K., Sawada T., Hashimoto H., Jimba M. (2019). Health-care disparities for foreign residents in Japan. Lancet.

[49] Kollar E., Buyx A. (2013). Ethics and policy of medical brain drain: a review. Swiss Med Wkly.

[50] World Health Organization (1986). First international conference on health promotion, Ottawa. https://www.who.int/teams/health-promotion/enhanced-wellbeing/first-global-conference.

[51] Conrad P. (2005). The shifting engines of medicalization. J Health Soc Behav.

[52] Gilmore A.B., Fabbri A., Baum F. (2023). Defining and conceptualising the commercial determinants of health. Lancet.

[53] Lenton T.M., Boulton C.A., Scheffer M. (2022). Resilience of countries to COVID-19 correlated with trust. Sci Rep.

[54] Árnason V. (2013). Scientific citizenship in a democratic society. Public Underst Sci.

[55] Sanders G.D., Neumann P.J., Basu A. (2016). Recommendations for conduct, methodological practices, and reporting of cost-effectiveness analyses: second panel on cost-effectiveness in health and medicine. JAMA.

[56] Montagu D., Goodman C. (2016). Prohibit, constrain, encourage, or purchase: how should we engage with the private health-care sector?. Lancet.

[57] Brownlee S., Chalkidou K., Doust J. (2017). Evidence for overuse of medical services around the world. Lancet.

[58] Delfraissy J.-F., Horgan M., Mølbak K. (2024). Scientific advisory councils in the COVID-19 response. Lancet.

[59] Bavel J.J.V., Baicker K., Boggio P.S. (2020). Using social and behavioural science to support COVID-19 pandemic response. Nat Hum Behav.

[60] Ishizumi A., Kolis J., Abad N. (2024). Beyond misinformation: developing a public health prevention framework for managing information ecosystems. Lancet Public Health.

[61] OECD (2022).

[62] Ettelt S., Mays N., Allen P. (2015). The multiple purposes of policy piloting and their consequences: three examples from national health and social care policy in England. J Soc Policy.

[63] Khin Y.P., Owusu F.M., Nawa N., Surkan P.J., Fujiwara T. (2025). Barriers and facilitators for healthcare access among immigrants in Japan: a mixed methods systematic review and meta-synthesis. Lancet Reg Health West Pac.

[64] Campanella P., Vukovic V., Parente P., Sulejmani A., Ricciardi W., Specchia M.L. (2016). The impact of public reporting on clinical outcomes: a systematic review and meta-analysis. BMC Health Serv Res.

[65] Patient Reported Outcome Measures (PROMs) NHS engl. Digit. https://digital.nhs.uk/data-and-information/data-tools-and-services/data-services/patient-reported-outcome-measures-proms.

[66] Find healthcare providers: compare care near you | Medicare. https://www.medicare.gov/care-compare.

[67] Romanello M., Walawender M., Hsu S.-C. (2024). The 2024 report of the Lancet Countdown on health and climate change: facing record-breaking threats from delayed action. Lancet.

[68] Golden C.D., Childs M.L., Mudele O.E. (2025). Climate-smart public health for global health resilience. Lancet Planet Health.

[69] Morse D.F., Sandhu S., Mulligan K. (2022). Global developments in social prescribing. BMJ Glob Health.

[70] Giraudeau C., Bailly N. (2019). Intergenerational programs: what can school-age children and older people expect from them? A systematic review. Eur J Ageing.

[71] Wagstaff A., Flores G., Hsu J. (2018). Progress on catastrophic health spending in 133 countries: a retrospective observational study. Lancet Glob Health.

[72] World Health Organization Earmarked tobacco taxes: lessons learnt from nine countries. https://www.who.int/publications/i/item/9789241515825.

[73] Milly D.J. (2017). Seeking skilled care workers: political explanations for Japan's emergent structure of care work migration. Asia Pac J Soc Work Dev.

[74] Laurence J., Igarashi A., Ishida K. (2022). The dynamics of immigration and anti-immigrant sentiment in Japan: how and why changes in immigrant share affect attitudes toward immigration in a newly diversifying society. Soc Forces.

[75] van der Linden S., Roozenbeek J. (2024). “Inoculation” to resist misinformation. JAMA.

