# Supplementary material for: Japan's health system toward 2040: structural challenges and a renewed social contract
Source: Lancet Reg Health West Pac. 2026 Jul 15;72:101920. doi: 10.1016/j.lanwpc.2026.101920 (PMC13478816; doi:10.1016/j.lanwpc.2026.101920)
Supplement: Supplementary file 1 [file mmc1.pdf]

Supplementary Appendix 1

Japan’s health system toward 2040: structural challenges and a renewed social contract

Contents

Supplementary Method: Expert working group consultation: procedures and survey instruments
Supplementary Table 1: The institutional basis of insularity: Japan’s foreign workforce policies for care workers
Supplementary Figure 1: Annual number of foreign care workers in Japan by visa pathway (2017–2024)

Supplementary Method: Expert working group consultation: procedures and survey instruments

Section 1. Working group composition

The working group had 25 members, including academics, diplomats, and development practitioners from Japan and the Western Pacific region, with attention to a mix of senior and early-career members and gender balance. The following table shows the composition by institutional type and regional background.

Working group composition

Dimension	Category	n
Institutional type	Academic institutions	15
	Multilateral / intergovernmental organisations and regional think tanks	4
	Policy implementation bodies and government-affiliated institutions	6
Regional background	Japan	19
	Other countries (China, Singapore, Thailand, Myanmar, Nepal, Germany)	6

Section 2. Consultation procedure

2.1 Two-round design rationale

The consultation ran in two rounds with different purposes. In Round 1, the Challenge Mapping round, we identified structural challenges confronting Japan's health system and its global health strategy. Round 2, the Goal and Action Mapping round, took place after the Round 1 results had been synthesised and shared with the working group. In Round 2, we set out forward-looking goals and concrete recommendations linked to the Round 1 challenges. Running the rounds in sequence ensured that the Round 2 recommendations rested on the diagnosis produced in Round 1.

2.2 Round 1 procedure and response format

Round 1 took place in September 2025. We provided up to three challenges in each of three dimensions: the international dimension covered global health financing, governance, and institutional design. The domestic dimension covered issues rooted in Japan's own systems and operations. The interface dimension covered the policy space between international initiatives and domestic systems. Entries were capped at 150 words, and we added a brief note on background or rationale. Section 3 reproduces the full Round 1 instrument.

2.3 Round 2 procedure and response format

Round 2 took place in November 2025 after the synthesised Round 1 results had been shared with the working group. We provided up to three goals and up to three actions, each capped at 150 words. For every entry, we also ticked one or more of six boxes indicating which of the Round 1 challenges the entry addressed. The six boxes were labelled A-1 to A-3 for global health and B-1 to B-3 for domestic health. Section 4 reproduces the full Round 2 instrument.

2.4 Coding and consensus procedure

Responses were analysed using inductive thematic analysis, through six iterative stages: familiarisation, initial coding generated directly from the data, theme generation, theme review, theme definition and naming, and assessment of thematic convergence. One of us conducted the initial coding of responses, and two others subsequently reviewed and verified the coding. We then discussed the consolidated themes and their interpretation to reach consensus. Codes were then grouped into higher-order themes. In Round 1, themes fell into the three dimensions used in the instrument, namely international, domestic, and interface, and were grouped further into six structural challenge categories: A-1 to A-3 for global health and B-1 to B-3 for domestic health. The three B categories correspond to the three structural challenges diagnosed in Table 1 of the main text. In Round 2, each response was first classified as either a goal, meaning a desired end-state, or an action, meaning a step for reaching that state, and coded on that basis. The resulting codes were consolidated into the goal-action matrix in Table 2 of the main text, which links each diagnosed challenge to the strategic recommendations proposed for it.

2.5 Working group meetings

The working group convened twice in plenary during the consultation. The first meeting, held in October 2025 between the two rounds, reviewed the synthesised Round 1 findings and aligned on the framing for Round 2. The second meeting, held in December 2025 after Round 2, reviewed the synthesised goals, actions, and the resulting goal–action matrix, and provided an opportunity to refine the diagnosed challenges and the wording of the recommendations presented in the main text.

Section 3. Round 1 survey instrument: Challenge Mapping Survey

The following reproduces the Round 1 survey instrument as administered to authors in September 2025.

Guidelines for Response

Please categorise your challenges under the three themes below. Within your professional scope, please provide up to three challenges, with each entry not exceeding 150 words. Where possible, please include a brief explanation of the background or rationale.

- **International Dimension:** Challenges related to global health financing, governance, and institutional design, where Japan faces difficulties or where Japan's role is at stake amid international developments.
- **Domestic Dimension:** Challenges rooted in Japan's own systems and operations, such as demographic change, healthcare financing, workforce shortages, and system reforms.
- **Interface between International and Domestic Dimensions:** Challenges arising at the policy interface, such as insufficient translation of international initiatives into domestic systems or limited utilisation of domestic knowledge and reforms to build international trust, both of which hinder a two-way cycle.

Examples

Clear challenges

- Declining global health financing raises concerns about the sustainability of health system strengthening in low- and middle-income countries.
- Institutional and financing structures in Japan are under increasing strain, raising concerns about the long-term sustainability of universal health coverage.
- While the international circulation of healthcare professionals is advancing, it has not sufficiently alleviated domestic workforce shortages.

Unclear challenges

- "How can more funding be mobilised?" (framed as a question rather than a challenge)
- "The UHC Knowledge Hub should be strengthened" (expressed as a solution rather than a challenge)
- "Perhaps AI could be used" (a proposal without a clearly defined challenge)

The challenges collected will provide the starting point for subsequent deliberations on "vision" and "action."

Response Page (Challenge Mapping Survey)

Within your professional scope, please list up to three challenges for each category below. Each entry should not exceed 150 words. Where possible, please provide a concise explanation of the background or rationale.

1. International Dimension

Challenges related to global health financing, governance, and institutional design, where Japan faces difficulties or where Japan's role is at stake amid international developments.

Challenge 1:

[response field]

Challenge 2:

[response field]

Challenge 3:

[response field]

2. Domestic Dimension

Challenges inherent to Japan's own systems and operations, such as population ageing, healthcare financing, workforce shortages, and system reforms.

Challenge 1:

[response field]

Challenge 2:

[response field]

Challenge 3:

[response field]

3. Interface between International and Domestic Dimensions

Challenges arising at the policy interface, such as insufficient translation of international initiatives into domestic systems or limited utilisation of domestic knowledge and reforms to build international trust, both of which hinder a two-way cycle.

Challenge 1:

[response field]

Challenge 2:

[response field]

Challenge 3:

[response field]

Optional Free-Entry Section

Please add any additional comments or related points you consider relevant.

Section 4. Round 2 survey instrument: Goal and Action Mapping Survey

The following reproduces the Round 2 survey instrument as administered to authors in November 2025.

Guidelines for Response: Defining "Goals" and "Actions"

To ensure clarity and consistency, please adhere to the following definitions, which distinguish between the desired future state (Goal) and the strategic steps to get there (Action).

Part 1: GOALS (The “What”)
Definition: A “Goal” (which we previously termed “Vision”) defines “What is the desired future state we should aim for?”.
Explanation: This is not a specific, granular solution. Instead, it should be a compelling, high-level depiction of the new paradigm or the ideal state we want to achieve once the Structural Challenges have been overcome. It describes the destination.

Examples of a GOAL:
Clear Examples (what we want):
“A resilient and adaptive ‘learning’ health system that institutionalises the integration of global best practices.”
“A proactive and consistent Japanese global health posture that shapes new international norms on health and security.”
Unclear Examples (what to avoid):
“Japan should establish an independent HTA body.” (This is an Action.)
“Japan’s leadership is too episodic.” (This is a Challenge.)

Part 2: ACTIONS (The “How”)
Definition: An “Action” defines “How do we get there?”.
Explanation: This is a concrete, high-level strategic recommendation required to move from the current Challenge to the desired Goal. It is the vehicle, not the destination.

Examples of an ACTION:
Clear Examples (what we want):
- “Establish a permanent, cross-ministerial body linking health to economic security to enable proactive norm-setting.”
- “Shift resource allocation from an acute, hospital-centric model to integrated, community-based care for multi-morbidity.”

Unclear Examples (what to avoid):
“Japan’s system should be more sustainable.” (This is a Goal.)
“The fee-for-service model is inefficient.” (This is a Challenge.)

Response Page (Goal and Action Mapping Survey)

Within your professional scope, please list up to three (3) Goals and up to three (3) Actions. Each entry should not exceed 150 words. For each entry, please check the box(es) corresponding to the primary Structural Challenge(s) it addresses.

Part 1: GOALS (The "What")

What is the desired future state we should aim for?

Goal 1:

[response field; max 150 words]

Related Structural Challenge(s) (please check all that apply):

☐ A-1: The Shifting Global Health Architecture and the Crisis of Solidarity
☐ A-2: Japan's Fragmented Policy and the "Episodic" Nature of its Leadership
☐ A-3: Breakdown in the Circular Model: Missed Opportunities for Mutual Growth
☐ B-1: A Systemic Mismatch with a New Era of Health Challenges
☐ B-2: An Outdated Infrastructure Inhibiting a Paradigm Shift
☐ B-3: Insularity and Failure to Leverage Global Interdependence

Goal 2:

[response field; max 150 words]

Related Structural Challenge(s) (please check all that apply):

☐ A-1: The Shifting Global Health Architecture and the Crisis of Solidarity
☐ A-2: Japan's Fragmented Policy and the "Episodic" Nature of its Leadership
☐ A-3: Breakdown in the Circular Model: Missed Opportunities for Mutual Growth
☐ B-1: A Systemic Mismatch with a New Era of Health Challenges
☐ B-2: An Outdated Infrastructure Inhibiting a Paradigm Shift
☐ B-3: Insularity and Failure to Leverage Global Interdependence

Goal 3:

[response field; max 150 words]

Related Structural Challenge(s) (please check all that apply):

☐ A-1: The Shifting Global Health Architecture and the Crisis of Solidarity
☐ A-2: Japan's Fragmented Policy and the "Episodic" Nature of its Leadership
☐ A-3: Breakdown in the Circular Model: Missed Opportunities for Mutual Growth
☐ B-1: A Systemic Mismatch with a New Era of Health Challenges
☐ B-2: An Outdated Infrastructure Inhibiting a Paradigm Shift

☐ B-3: Insularity and Failure to Leverage Global Interdependence

Part 2: ACTIONS (The "How")

What concrete, high-level strategic recommendations are necessary to achieve these Goals?

Action 1:

[response field; max 150 words]

Related Structural Challenge(s) (please check all that apply):

☐ A-1: The Shifting Global Health Architecture and the Crisis of Solidarity

☐ A-2: Japan's Fragmented Policy and the "Episodic" Nature of its Leadership

☐ A-3: Breakdown in the Circular Model: Missed Opportunities for Mutual Growth

☐ B-1: A Systemic Mismatch with a New Era of Health Challenges

☐ B-2: An Outdated Infrastructure Inhibiting a Paradigm Shift

☐ B-3: Insularity and Failure to Leverage Global Interdependence

Action 2:

[response field; max 150 words]

Related Structural Challenge(s) (please check all that apply):

☐ A-1: The Shifting Global Health Architecture and the Crisis of Solidarity

☐ A-2: Japan's Fragmented Policy and the "Episodic" Nature of its Leadership

☐ A-3: Breakdown in the Circular Model: Missed Opportunities for Mutual Growth

☐ B-1: A Systemic Mismatch with a New Era of Health Challenges

☐ B-2: An Outdated Infrastructure Inhibiting a Paradigm Shift

☐ B-3: Insularity and Failure to Leverage Global Interdependence

Action 3:

[response field; max 150 words]

Related Structural Challenge(s) (please check all that apply):

☐ A-1: The Shifting Global Health Architecture and the Crisis of Solidarity

☐ A-2: Japan's Fragmented Policy and the "Episodic" Nature of its Leadership

☐ A-3: Breakdown in the Circular Model: Missed Opportunities for Mutual Growth

☐ B-1: A Systemic Mismatch with a New Era of Health Challenges

☐ B-2: An Outdated Infrastructure Inhibiting a Paradigm Shift

☐ B-3: Insularity and Failure to Leverage Global Interdependence

Optional Free-Entry Section

Please add any additional comments or related points you consider relevant.

Supplementary Table 1: The institutional basis of insularity: Japan's foreign workforce policies for care workers. This table compares the four major visa-based pathways through which foreign care workers are admitted to Japan's health and long-term care sectors. For each scheme, we describe its legal basis and stated policy purpose alongside the structural challenges that undermine effective workforce integration. The comparison illustrates how Japan's fragmented approach has historically framed foreign workers as temporary labour supplements rather than long-term partners in system sustainability, thereby constraining opportunities for reciprocal skills transfer and circular innovation.

Scheme	Legal basis and policy purpose	Challenges
Economic Partnership Agreement (EPA)	• Designed not as a labour shortage countermeasure, but as a special diplomatic exception to accept "candidates" based on bilateral treaties aimed at strengthening economic partnerships (currently with Indonesia, the Philippines, and Viet Nam).[1]<br>• Launched in 2008 (Indonesia), followed by the Philippines (2009) and Viet Nam (2014), based on specific bilateral Economic Partnership Agreements and special public notices under the Immigration Control Act.	• **Language-based systemic barriers:** The requirement to pass the national licensure examination for care workers and nurses (which must be taken in Japanese) acts as a formidable barrier and failure to pass within the permitted period ultimately precludes continued EPA-based residence, though since 2012 a one-year extension has been available and transition to Specified Skilled Worker status offers an alternative pathway.[2]<br>• **Inefficient resource allocation:** Despite high government and institutional investment in language training and matching, the system yields a low number of fully licensed professionals, limiting its scalability.[1]
Technical Intern Training Program (TITP)	• Officially designates "international contribution" via technology transfer to developing countries, explicitly denying its role as a means to adjust labour supply and demand.<br>• Originally established in 1993; currently operated under the Act on Proper Technical Intern Training and Protection of Technical Intern Trainees (Japanese statute governing the TITP framework) (enforced in 2017).[3]	• **Career discontinuity:** The program is structurally designed around time-limited stays with an expectation of return to the country of origin, and does not envisage long-term settlement or career continuity within Japan.<br>• **Structural vulnerability:** The restriction on changing employers binds workers to specific institutions, creating power imbalances that have been subject to sustained international criticism

	• The Employment for Skill Development program (effective April 2027) will replace the TITP, shifting from the “international contribution” framing to explicit labour-force objectives, formalising career pathways, and introducing regulated job transfers.[4] Although this is a meaningful advance, the new program is still designed mainly to fill Japan’s domestic workforce gap, and provisions for skills transfer back to source countries remain limited.	regarding labour rights and worker protection.[5]
Specified Skilled Worker (SSW)	• Represents a historic policy shift by officially recognising the need for foreign workers to address severe domestic labour shortages in specific industries, aiming to secure work-ready personnel.<br>• Launched in 2019 based on the amendment to the Immigration Control and Refugee Recognition Act (Japan’s principal immigration statute), creating a new residence status distinct from professional visas.[6]	• **Integration dead-end:** While entry is permitted, pathways to permanent residency and professional advancement remain structurally limited unless national licensure requirements are met.<br>• **Social isolation:** SSW care workers are prohibited from bringing family members. Moreover, while other SSW sectors offer an upgraded status permitting family accompaniment and long-term stay, nursing care is explicitly excluded – the only route to long-term settlement for care workers remains obtaining national certification and transitioning to the dedicated 'Nursing Care' residence status. This compounds social isolation and reduces the appeal of Japan as a long-term destination.[6,7]
Domestic Education Pathway: Student Visa to 'Nursing Care' Visa	• Aims to retain highly specialised personnel trained at Japanese vocational facilities.<br>• Because graduates obtain credentials equivalent to those of domestic students, this pathway provides the most legally stable route to long-term residence among the four schemes.	• **Economic barriers:** The high cost of tuition and living expenses in Japan creates a significant entry barrier for students from the Global South, limiting this “gold standard” route to a wealthy minority.<br>• **High-stakes risk:** Students face a double burden of mastering academic Japanese and passing the national certification examination

	• Established in 2017 as the "Nursing Care" residence status,[8] based on amendments to the Immigration Control Act and the Certified Social Worker and Certified Care Worker Act (Japan's principal statute governing professional care qualifications).	for care workers; a transitional measure (currently in effect through FY 2026) allows training facility graduates to register as certified care workers for five years without passing the national examination;[9] however, the transitional measure's continuation beyond FY2026 remains uncertain, and the long-term risk of qualification and residency loss persists.

Supplementary Figure 1: Foreign care workers in Japan by visa pathway (2017–2024)

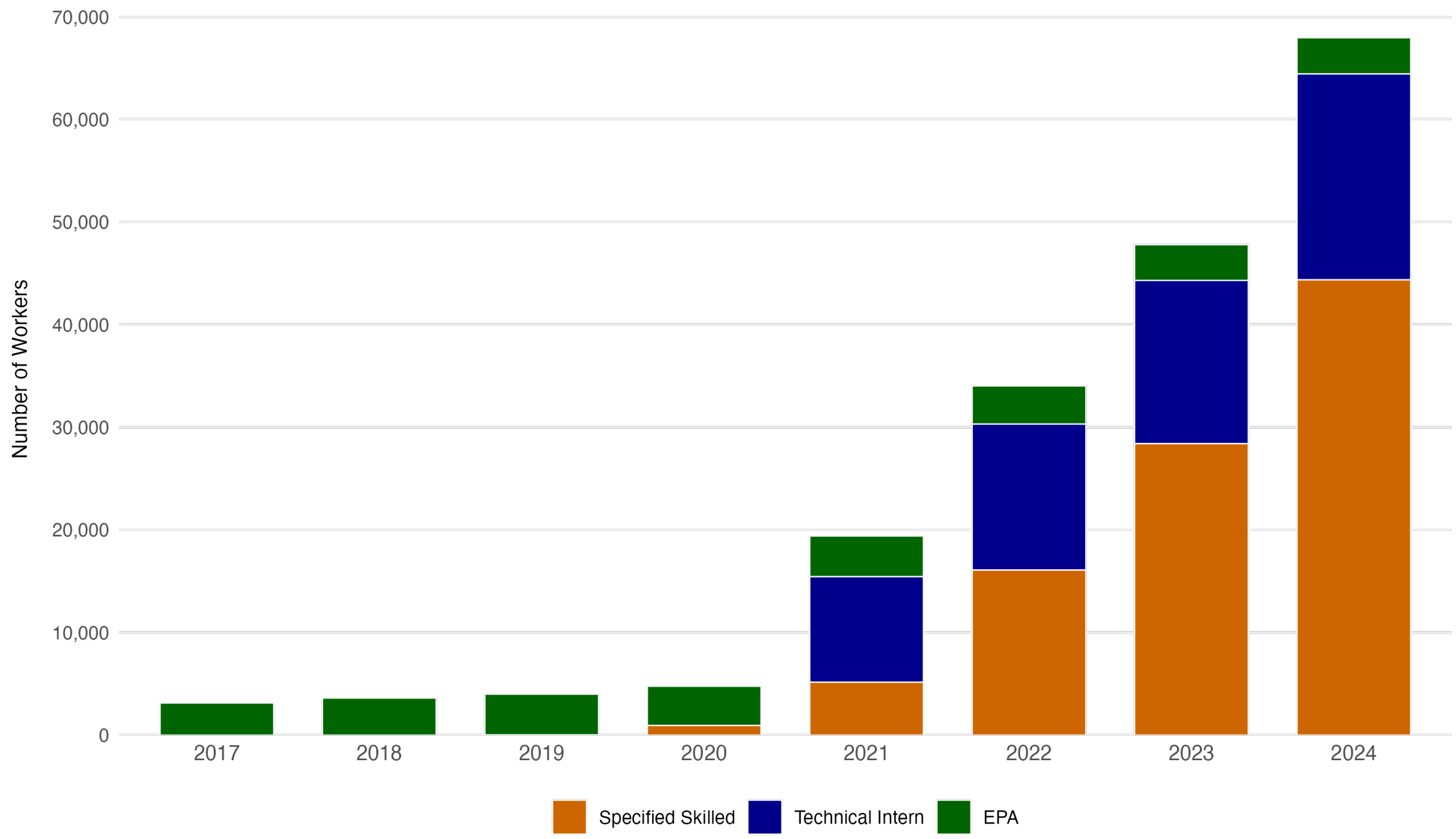

This figure was compiled by the authors using publicly available administrative data from the sources listed below. The stacked bar chart displays the total number of foreign care workers employed in Japan as of each year across three major visa pathways from 2017 to 2024. The y-axis represents the total number of workers; the x-axis represents calendar year. Green bars indicate workers under the Economic Partnership Agreement (EPA); blue bars indicate those under the Technical Intern Training Program (TITP); orange bars indicate Specified Skilled Worker (SSW) visa holders. The Domestic Education Pathway (whereby foreign nationals obtain a care worker qualification through Japanese vocational schools and then transition to a dedicated 'Nursing Care' residence status) is not included due to the absence of consolidated annual statistics. SSW numbers have expanded rapidly since the pathway opened in 2019, becoming the largest single source of foreign care workers by 2024. EPA intake has remained roughly flat, and TITP numbers have continued to rise, despite long-standing concerns about worker protections. Note that TITP data were first published by the Immigration Services Agency for year-end 2021.

Data source: Immigration Services Agency of Japan.[10–12]

References

1. OECD. Recruiting Immigrant Workers: Japan 2024. 2024. https://www.oecd.org/en/publications/recruiting-immigrant-workers-japan-2024_0e5a10e3-en.html (accessed May 1, 2026).
2. Ministry of Health, Labour and Welfare. Publication of national examination score thresholds for extension of stay of foreign nurse and certified care worker candidates under Economic Partnership Agreements (EPAs) [Japanese]. 2023. https://www.mhlw.go.jp/stf/newpage_32212.html (accessed May 1, 2026).
3. Japan International Trainee & Skilled Worker Cooperation Organization (JITCO). Technical Intern Training Program [Japanese]. 2025. https://www.jitco.or.jp/ja/regulation/ (accessed May 1, 2026).
4. Immigration Services Agency of Japan. Overview of the Development Employment System (Revised December 2025) [Japanese]. 2025. https://www.moj.go.jp/isa/content/001452485.pdf (accessed May 1, 2026).
5. U.S. Department of State. 2021 Trafficking in Persons Report: Japan. 2021. https://www.ecoi.net/en/document/2055211.html (accessed May 1, 2026).
6. Ministry of Foreign Affairs of Japan. What is the SSW? https://www.mofa.go.jp/mofaj/ca/fna/ssw/us/overview/ (accessed May 1, 2026).
7. Japan International Trainee & Skilled Worker Cooperation Organization (JITCO). Expansion of industrial fields for SSW (ii) - Japanese cabinet decisions on 9th June 2023. https://www.jitco.or.jp/en/news/article/1423/ (accessed May 1, 2026).
8. Immigration Services Agency of Japan. Major Policies on Immigration Control Administration. 2023. https://www.moj.go.jp/isa/content/930003501.pdf (accessed May 1, 2026).
9. Social Welfare Promotion and National Examination Center (SSSC). Transitional measures registration procedures for graduates of certified care worker training institutions from April 1, 2017 to March 31, 2027. https://www.sssc.or.jp/touroku/info/info_keika.html (accessed May 1, 2026).
10. Portal Site of Official Statistics of Japan (e-Stat). Statistics on Foreign Residents [Japanese]. https://www.e-stat.go.jp/stat-search/files?page=1&layout=datalist&toukei=00250012&tstat=000001018034&cycle=1&tclass1=000001060399&cycle_facet=tclass1&tclass2val=0 (accessed May 1, 2026).
11. Immigration Services Agency. Publication of the number of foreign nationals with specified skills. https://www.moj.go.jp/isa/applications/ssw/nyuukokukanri07_00215.html?hl=en (accessed May 1, 2026).
12. Immigration Services Agency. Public Information (List of Supervising Organizations, Administrative Disciplinary Actions, Number of Missing Persons, etc.).

https://www.moj.go.jp/isa/applications/titp/nyuukokukanri07_00138.html?hl=en (accessed May 1, 2026).
