# Supplementary material for: Japan's health system toward 2040: structural challenges and a renewed social contract
Source: Lancet Reg Health West Pac. 2026 Jul 15;72:101920. doi: 10.1016/j.lanwpc.2026.101920 (PMC13478816; doi:10.1016/j.lanwpc.2026.101920)
Supplement: Supplementary file 2 [file mmc2.docx]

**Supplementary Appendix**

Translated abstract in Japanese

Japan’s health system toward 2040: structural challenges and a renewed social contract

*Editor’s note: This translation in Japanese was submitted by the authors and we reproduce it as supplied. It has not been peer reviewed. Our editorial processes have only been applied to the original abstract in English, which should serve as reference for this manuscript.*

日本の国民皆保険制度は、健康長寿の実現を支えてきた。しかし現在、この制度は、広く認識されている構造的な圧力に直面しながらも、その中核となる仕組みを十分に見直せていないという制度的停滞に陥っている。本稿では、日本および西太平洋地域の25名の専門家からなる学際的ワーキンググループを通じて、政策停滞を持続させる三つの相互に関連した構造的課題を特定した。第一に、出来高払いのインセンティブ、資源配分の硬直性、未成熟な医療技術評価に支えられた、量重視・病院中心の医療提供体制である。第二に、エビデンスに基づくガバナンスを弱める、分断されたデータ基盤と公衆の信頼の低下である。第三に、国際的な相互依存関係の活用を制約する制度的な閉鎖性である。 我々は、三つの目標からなる「Vision 2040」の枠組みを提案する。すなわち、日常生活機能とウェルビーイングを重視する、開かれた地域基盤型エコシステムへの移行、ガバナンスの基盤としての信頼と科学的独立性の制度化、そして、日本が地域の保健医療ネットワークに貢献すると同時にそこから学び、国際的な学びと国内の価値評価を統合する「循環型イノベーション」を通じた公平性の確保である。九つの提言は、ガバナンス改革、データ統合、社会的価値を反映した診療報酬体系の再設計、相互的な人材交流パートナーシップに向けたロードマップを示す。国民皆保険制度を支える社会的な合意を再構築することは、高齢化が進む西太平洋地域の社会における持続可能性の中核的課題である。
